# The ACPGBI recommends pause for reflection on transanal total mesorectal excision

**DOI:** 10.1111/codi.15143

**Published:** 2020-07-24

**Authors:** N. S. Fearnhead, A. G. Acheson, S. R. Brown, L. Hancock, A. Harikrishnan, S. B. Kelly, C. A. Maxwell‐Armstrong, P. M. Sagar, S. Siddiqi, C. J. Walsh, J. M. D. Wheeler, J. F. Abercrombie, Association of Coloproctology of Great Britain, Ireland (ACPGBI) Executive, Getting It Right First Time (GIRFT)

**Affiliations:** ^1^ Cambridge University Hospitals NHS Foundation Trust Cambridge UK; ^2^ Nottingham University Hospitals NHS Trust Nottingham UK; ^3^ Sheffield Teaching Hospitals NHS Foundation Trust Sheffield UK; ^4^ Wythenshawe Hospital Manchester University NHS Foundation Trust Manchester UK; ^5^ Northumbria Healthcare NHS Foundation Trust Newcastle upon Tyne UK; ^6^ Leeds Teaching Hospitals NHS Trust Leeds West Yorkshire UK; ^7^ Broomfield Hospital Mid Essex Hospital NHS Trust Chelmsford Essex UK; ^8^ Arrowe Park Hospital Wirral University Teaching Hospital NHS Foundation Trust Wirral Merseyside UK

**Keywords:** Ileoanal pouch, outcomes, rectal cancer, total mesorectal excision, transanal

The Association of Coloproctology of Great Britain and Ireland (ACPGBI) has noted with concern the results of transanal total mesorectal excision (TME) procedures reported by the Norwegian Colorectal Cancer Registry, recently published in the *British Journal of Surgery* [1]. The major cause for alarm has been the unexpectedly high rate of early multifocal local pelvic recurrence. These findings have led to a national moratorium on transanal TME for rectal cancer in Norway.

Other published causes for concern about this novel approach to TME include a relatively high incidence of urethral injuries occurring during both the learning curve and in established practice [2], irrespective of completion of appropriate accredited training [3], an unexpected incidence of carbon dioxide embolism [4] and high rates of morbidity during the learning curve, even within a structured national training programme [5]. Rates of anastomotic leakage appear reasonable in most retrospective series, but were 16.5% in a Dutch study examining the experience of transanal TME at two tertiary Dutch centres [6] and statistically higher than other approaches in the Norwegian registry report [1]. Although a systematic review of retrospective and potentially biased case series reported anastomotic leakage rates better than laparoscopic TME [7], meta‐analysis of the small number of patients included in randomized trials comparing the various approaches to TME indicated comparable rates [8].

Innovation in surgery has undoubted potential to enhance patient care and outcomes. The ACPGBI supports rigorous assessment of new techniques within the IDEAL framework as fundamental for assessing both potential patient benefits and harms. Following the publication of the National Institute for Health and Care Excellence (NICE) Interventional Procedures Guidance IPG514 on transanal TME of the rectum in 2015, the ACPGBI issued guidance to its members (https://www.acpgbi.org.uk/news/acpgbi‐position‐statement‐on‐transanal‐total‐mesorectal‐excision‐tatme/). Emphasis was placed on appropriate training, the need for two consultants to operate together, patient consent specifically about use of the new approach and mandatory contribution of data to the national transanal TME registry. Members of our organization subsequently led the international consensus to develop a structured framework for introduction of transanal TME, including high standards for proctors, mentees and data collection [9].

The ACPGBI subsequently coordinated an industry‐sponsored training and proctoring programme with strict institutional criteria based on number of rectal cancer resections, and surgeon specifications based on proficiency in laparoscopic rectal cancer surgery and prior cadaveric training. The first phase proctored surgeons from six centres and the second phase has, to date, covered four centres. Cases have been recorded in the UK Transanal TME Registry which is now incorporated in the International Transanal TME Registry. It is worth noting that only three‐quarters of UK‐based surgeons trained in transanal TME go on to establish it in their own institution [10].

The Transanal TME Registry has recently reported short‐term outcomes for 513 patients (364/513, 71% had rectal cancer) undergoing transanal TME in 42 UK institutions between 2013 and 2018 [10]. Twenty‐eight of the 42 hospitals performed fewer than 10 procedures. Pathological criteria on the resected TME specimen were used as a surrogate marker of oncological outcome; satisfactory pathology was noted in 92.8% of cases and the R1 rate for involved circumferential resection margin (CRM) was around 4%. Significant complications were reported in 13.4% of cancer patients. No long‐term UK data have yet been reported on either recurrence or survival.

Similar results for involved CRM rates are reported in retrospective combined institutional series from the Netherlands [6] and the USA [11]. Short‐term outcomes from the International Transanal TME registry were reported on 2653 patients undergoing a transanal TME approach for rectal cancer between 2014 and 2018 [12]. There was a similar R1 rate of 4%. Given the size of the data set, the study was able to define predictive factors for involved margins during transanal TME, including very low rectal cancers, anteriorly positioned tumours, T4 cancers, extramural vascular invasion and threatened/involved margins on staging MRI. Paradoxically, all these factors are often cited as the reasons why a transanal TME approach should be considered, especially in a male patient with a narrow pelvis and high body mass index.

Apart from the recent Norwegian report, there is a paucity of data on long‐term outcomes, and especially about survival and recurrence. The longest period of reported high‐quality evidence is from the Bordeaux randomized trial in 100 patients, which reported a 5‐year local recurrence rate of 3% and 5‐year disease‐free survival of 72%, very similar to the outcomes seen for patients undergoing laparoscopic TME in the 2008 to 2012 trial [13]. Combined long‐term retrospective experience from two Dutch tertiary centres on 159 patients indicated a local recurrence rate of 2% at 3 years and 4% at 5 years with a median time to local recurrence of 19.2 months, and overall survival rates of 83.6% and 77.3% at 3 and 5 years, respectively [14]. A smaller North American experience across two institutions of transanal TME in 54 patients reported a similar local recurrence rate of 3.9% at a median follow‐up of 2.3 years [11].

The recent Norwegian study included all 157 patients from seven institutions performing transanal TME in Norway between 2014 and 2018 [1]. The observed rate of local recurrence was 7.6% at a median follow‐up of 19.5 months, giving an estimated local recurrence rate of 11.6% at 2.4 years, compared with just 2.4% for all other TME patients in the Norwegian Colorectal Cancer Registry. Patients undergoing transanal TME had tumours with better prognosis and lower rates of neoadjuvant therapy, and so should have had lower recurrence rates than the national average. Two‐thirds of patients with recurrence were also noted to have unusual patterns of extensive or multifocal recurrence, limiting further treatment options. Three of seven Norwegian institutions had already abandoned the transanal TME approach after five cases prior to the review. While purse‐string failure to control shedding of cancer cells during rectal opening and manipulation during surgery is the likely cause of this phenomenon, the authors note that the recurrences were seen across all four high‐volume (range 32–57 cases) institutions, and so are likely to represent an inherent failing of the technical approach rather than a lack of appropriate training [1].

The COLOR III trial has been designed to assess the superiority of transanal TME over the laparoscopic approach for patients with mid and low rectal cancer [15], with involved CRM as the primary outcome and including local recurrence and survival as secondary outcomes during 5‐year follow‐up. The trial has been designed to deliver an admirably high‐quality transanal approach to TME [16] but the surveillance protocol only includes a single pelvic MRI at 3 years, which may be too late to detect the potential harms of multifocal recurrence observed in the Norwegian Registry.

International expert advisory guidance on indications, implementation and quality measures for transanal TME led by the European Society of Coloproctology will shortly be published (R. Hompes, pers. comm.). The guidance will sensibly advocate safe implementation of transanal TME through training and proctoring, appropriate case selection, standards for surgery, requirement for reporting data through national or international registries and consolidation of experience in high‐volume centres of excellence with a minimum of two colorectal surgeons per unit with the necessary expertise. Proponents of transanal TME have heeded lessons learned from the Norwegian Registry and are now advocating a double purse‐string technique with more extensive tumouricidal rectal washout (https://www.youtube.com/watch?v=u3HptXOi73g). This technique has been adopted in the COLOR III trial protocol [16] but also needs formal incorporation within UK and other national training programmes, as well as overt acceptance by established transanal TME programmes.

None of the colorectal community wishes to see a repetition of the early abandonment of laparoscopic colorectal cancer surgery in the early 1990s due to concerns about port site metastases, which later proved unfounded when appropriate extraction techniques were implemented [17]. Laparoscopic colorectal cancer resection has undoubtedly brought benefits for many patients, although case selection is important and subgroups such as patients with rectal cancer may not necessarily benefit from an oncological perspective [18, 19]. The transanal TME approach may yet offer potential benefit in terms of visualization of the distal mesorectum and potential for improved oncological and functional outcomes [8]. There may also be benefits for patients with benign disease such as those undergoing ileoanal pouch formation, where lack of adequate purse‐string control would not have the same potentially deleterious consequences. Nevertheless, rigorous assessment of new techniques is an ethical imperative, and must balance any perceived benefits with harms, even if only experienced by a minority [20].

While benefits and harms are being assessed for a new technique, properly informed patient consent is essential, and enhanced consent processes should be employed to ensure that patients are aware that a novel approach remains under validation.

Given the concerns raised, and while awaiting the results of the COLOR III trial, the ACPGBI has notified NICE of our concerns about the safety of transanal TME. Pending further guidance, the ACPGBI and Getting It Right First Time (GIRFT) are recommending a considered pause for re‐evaluation and consolidation of evidence on the transanal TME approach to resecting rectal cancer. Our recommendations are:

1. Temporary closure of the proctoring programme to new sites;
2. Extending the number of proctored cases from the current recommendation of 5–10 where sites are still completing the proctoring process;
3. Individual institutions to reconsider whether to continue transanal TME after review of local data, and subject to formal notification to local clinical governance authorities and permission of the medical director;
4. Transanal TME should only be carried out in institutions that undertake more than 40 rectal cancer resections (with rigorous exclusion of rectosigmoid cancer resections) each year, to allow sufficient ongoing experience to maintain surgical competency in the procedure;
5. Transanal TME should only be carried out in institutions that undertake more than 25 transanal rectal resections each year for rectal cancer and benign disease, to allow sufficient ongoing experience to maintain surgical competency in the technique;
6. Concentration of institutional experience in transanal TME by limiting performance of the procedure to two or three colorectal surgeons. Isolated practitioners are discouraged in order to ensure adequate local service delivery;
7. Use of procedure‐specific enhanced patient consent;
8. Mandatory entry of data about patient demographics, patient selection, operative details and outcomes on the International Transanal TME Registry;
9. Updating the international registry with long‐term oncological outcomes in patients who underwent resection for rectal cancer;
10. Independent review of the data held by the International Transanal TME Registry;
11. Assessment of the level of English and Welsh case ascertainment and data completeness in the International Transanal TME Registry through cross‐referencing with NHS Digital data;
12. Collection of transanal TME as a data item in the National Bowel Cancer Audit for England and Wales, and by the Scottish Colorectal Cancer networks.

The ACPGBI Executive accepts that some of these recommendations are based on pragmatic common sense rather than hard evidence, especially as the learning curve for safe independent practice of transanal TME has yet to be established. As transanal TME is only just moving from IDEAL stage 2 to stage 3 in terms of innovation assessment [21], it is inherent that the available evidence to guide recommendations is limited. Other recommendations are made in line with recent consensus guidance from 52 international experts who had performed a median of 25 (range 10–250) transanal TME procedures [9]. The annual institutional caseload of 25 transanal TME procedures is set at the lower limit of the 25 to >40 transanal rectal cancer resections recommended by the latest international transanal TME consensus, to which the ACPGBI contributed (R. Hompes, pers.comm.). Based on institutional caseloads in the 2019 National Bowel Cancer Audit Report, 85 institutions in England and Wales carried out 25 or more major rectal cancer resections during the financial year 2017–2018, with just 34 institutions performing more than 40 resections per year [22].

We believe that patients undergoing transanal TME for benign disease deserve the same level of scrutiny and protection from potential harms in using a novel technique, while appreciating that some colorectal surgeons will be offering transanal TME purely for benign disease. Our general recommendations for this patient group are identical to those for rectal cancer surgery, apart from the caveat about long‐term oncological outcomes.

The ACPGBI’s mission is to promote the prevention, care and cure of colorectal disease for the benefit of patients. While technical innovation may of course bring potential benefits, we have a duty of care to measure and report harms as well. Reflection will provide the opportunity to properly evaluate all aspects of transanal TME. It is our intention that lifting the pause on transanal TME training should be data‐driven (rather than time‐driven), pending the results of external review of the transanal TME registry data and further peer‐reviewed publications on potential harms and longer‐term oncological outcomes. The ACPGBI will examine its position on transanal TME once these reports are available, with the hope that further guidance can be issued within the next 3 years.
